# A Case of Total Excision of a Thrombosed-Venous Aneurysm in the Sural Vein

**DOI:** 10.3400/avd.cr.20-00054

**Published:** 2020-09-25

**Authors:** Masato Nishizawa, Kimihiro Igari, Masayuki Hirokawa, Nobuhisa Kurihara, Sotaro Katsui, Toshifumi Kudo, Hiroyuki Uetake

**Affiliations:** 1 Department of Specialized Surgery, Tokyo Medical and Dental University; 2 Ochanomizu Vascular & Vein Clinic

**Keywords:** venous aneurysm, sural vein, venous thromboembolism

## Abstract

Venous aneurysm (VA) is an uncommon vascular disease; however, VA, especially in the lower extremities, can lead to critical complications, such as pulmonary embolism (PE). We report a case with a VA located in the sural vein (SV), which did not lead to PE; however, it had the potential to cause PE. Therefore, we treated this VA by total excision. The popliteal vein (PV) is the most common VA location in the lower extremities, but SV is extremely rare. We should always be aware that, in addition to the PV, VAs may also occur in the SV.

## Introduction

Venous aneurysm (VA) is a rare vascular disease. Most VAs are located in the lower extremities, especially in the deep venous system.^1)^ The VA’s common pathological features are a focal defect of connecting tissue in the venous wall and reduction of smooth muscle cells, which can lead to focal dilatation of the venous wall.^1)^ VAs are normally located in the popliteal vein (PV),^2)^ and 24–51% of patients with popliteal VA (PVA) have shown symptomatic pulmonary embolism (PE).^3)^ Even though VAs can be located in other parts of the body, including the head, neck, and upper extremities, they are rarely related to PE. Thus, VAs in parts other than the lower extremities have been treated non-operatively.^4)^ Therefore, we should diagnose and treat VAs in the lower extremities correctly to prevent severe morbidity and mortality. Although we have already experienced some cases with PVA, VA in the sural vein (SV), namely a sural VA (SVA), is extremely rare. We could find only one case report written in Japanese^5)^; however, we have found no similar case reports written in English. We herein report the total excision of an SVA to prevent significant risk of mortality, especially due to PE.

## Case Report

**Figure 1:**
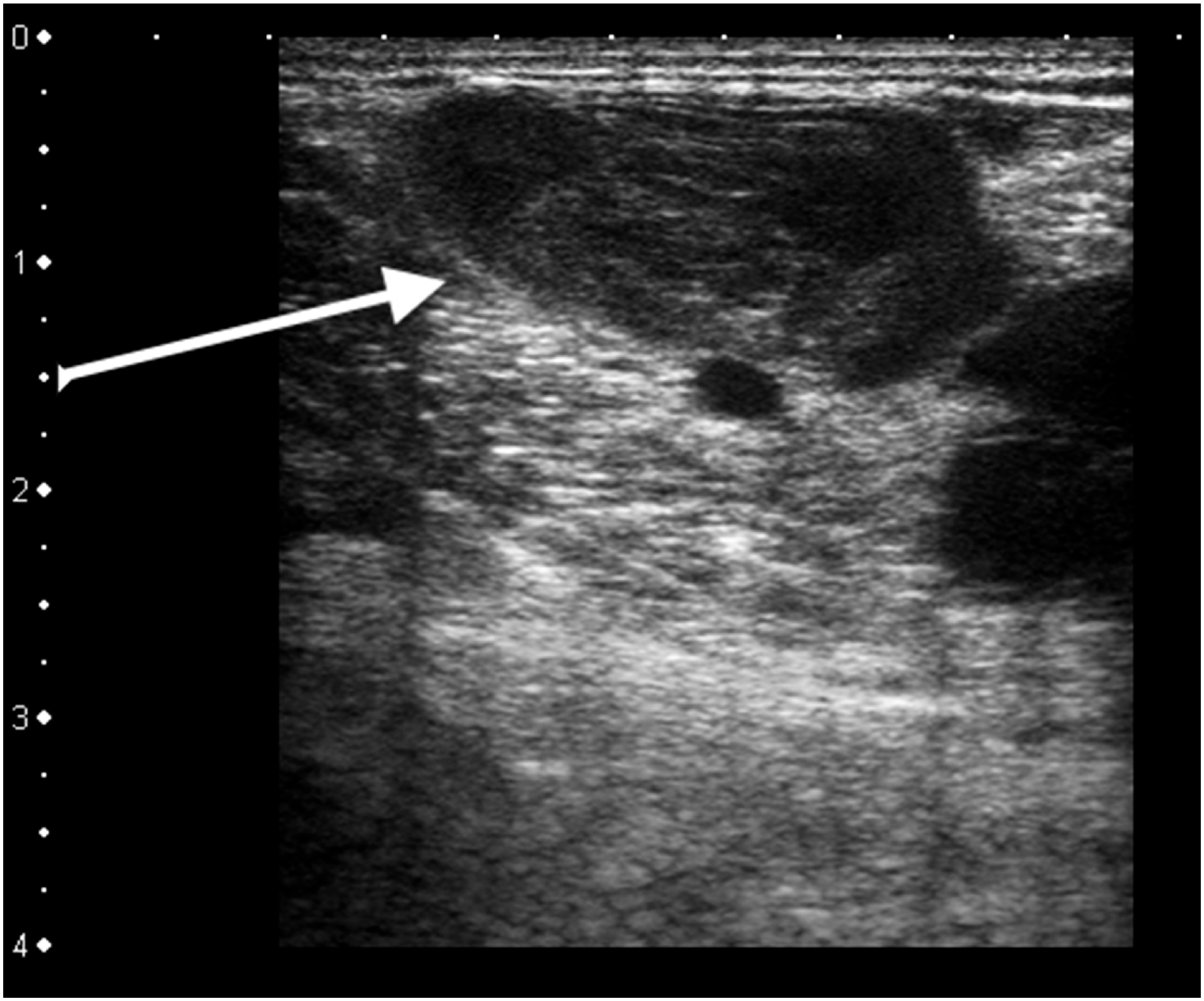
Fig. 1 Preoperative duplex ultrasonography showed a 24.3 mm ×20.4 mm saccular-type aneurysm in the sural vein with thrombus (white arrow).

**Figure 2:**
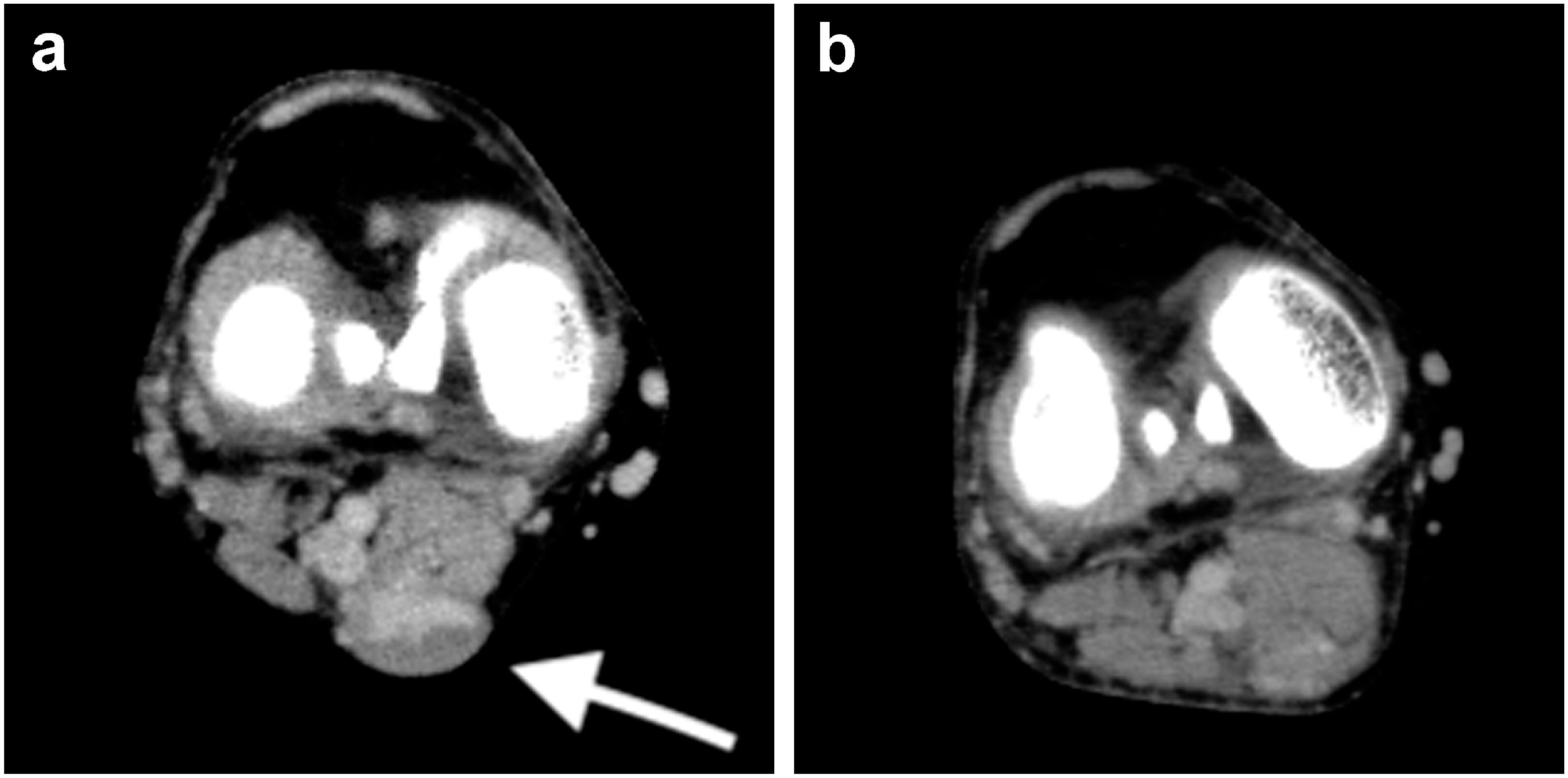
Fig. 2 (**a**) Preoperative contrast-enhanced computed tomography showed a saccular sural venous aneurysm on the right side with thrombus (white arrow). (**b**) Postoperative contrast-enhanced computed tomography showed no recurrent sural venous aneurysm in the right side of the popliteal fossa.

A 63-year-old man with no medical comorbidities or medications presented with a palpable popliteal mass. Duplex ultrasonography (DUS) revealed a 24.3 mm×20.4 mm saccular-type of VA with thrombus in the right side of the SV (**Fig. 1**). Contrast-enhanced computed tomography (CE-CT) showed the same aneurysm, without deep venous thrombosis (DVT), extending to the PV, or PE (**Fig. 2a**). The patient had no signs of venous disorders, such as edema, skin pigmentation, or ulceration. Thus, we diagnosed this patient with SVA and excised it to prevent further venous thromboembolic events, such as DVT or PE.

**Figure 3:**
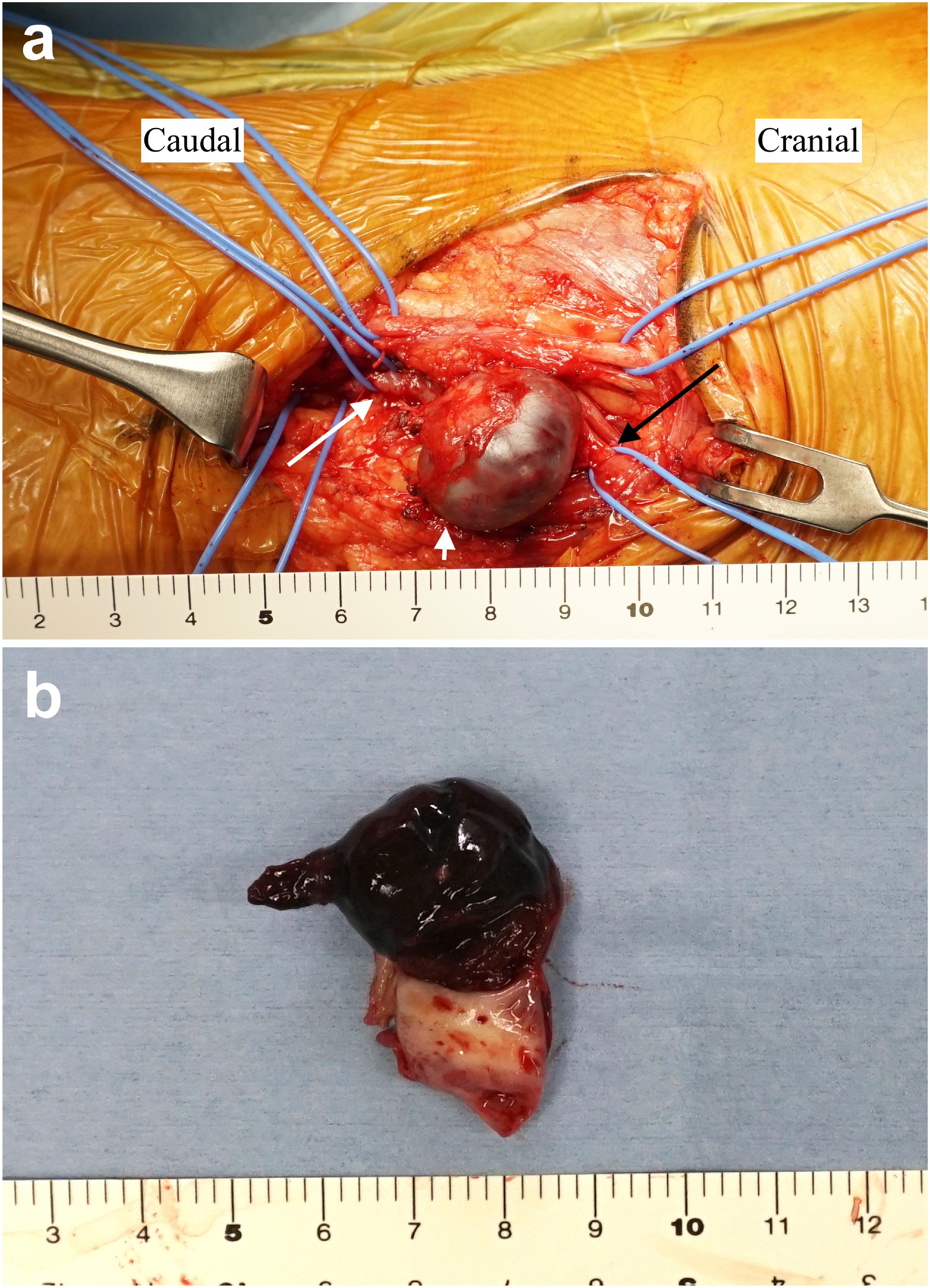
Fig. 3 (**a**) The intraoperative findings showed a sural venous aneurysm (SVA) (short white arrow), located near the popliteal vein. The black arrow indicated the junction between popliteal and sural veins, and long white arrow indicated the sural vein, the distal part of the SVA. (**b**) The resected venous aneurysm was 22 mm×21 mm with thrombus.

Under general anesthesia, we placed the patient in the prone position and made a lazy S-shaped incision in the posterior segment of the knee. The posterior approach requires care to avoid injuring the nerves, especially the tibial and sural nerves; therefore, we confirmed these nerves before controlling the SVA. The SVA was exposed, and the SV was controlled at both the proximal and the distal sites of the SVA (**Fig. 3a**). We did not need to maintain venous flow continuity in this SV; thus, after confirming the thrombus in the SVA, we totally excised the SVA and the thrombus (**Fig. 3b**). The pathological examination of the resected SVA showed a 30 mm×25 mm×20 mm-sized true aneurysm occluded by thrombi.

The postoperative course was uneventful, and the patient was discharged four days postoperatively with no oral anticoagulant treatments. A follow-up CE-CT scan and DUS one month postoperatively showed no recurrent VAs or venous thromboembolism (VTE) (**Fig. 2b**). Thus far, there have been no postoperative complications, including hematoma, deep venous insufficiency, or palsies of the tibial or peroneal nerves.

## Discussion

This report presented a case of a thrombosed-SVA that was treated by total excision. Even though we may be able to diagnose VAs by several different diagnostic modalities, such as DUS, these VAs are relatively uncommon. Thus, we should be aware of these vascular lesions, which might show specific symptoms, such as swelling, mass and pain.^1)^ As mentioned above, most VAs in the lower extremities, especially in the deep venous system, are located in the PVs. We reviewed the relevant literature but found no reports on SVA written in English. Thus, to our knowledge, this is the first report written in English on SVA.

Several indications for treating VAs in the lower extremities, including surgical resection, have been reported. Teter et al.^2)^ noted that VAs in the lower extremities, especially involving the deep venous system, should be treated whenever they are found to avoid critical VTE. On the other hand, Sessa et al.^6)^ reported that there were more saccular-type PVAs (72%) than fusiform-type PVAs (28%). They recommended that surgical treatment for VAs in the lower extremities should be based on the VA type and size. They recommended that all saccular-type VAs, with or without symptoms, and large-sized (>20 mm), fusiform-type VAs should be treated surgically. They also reported that saccular-type PVAs were found more frequently with intra-aneurysmal thrombus (40%) than fusiform-type PVAs (14%). Thus, saccular-type PVAs should be treated, despite their size, to prevent extension of the thrombus. Furthermore, Norimatsu et al. reported that thromboembolic complications are more frequent in large fusiform- and saccular-type VAs.^3)^ Furthermore, they pointed out that approximately 70% of PVAs contained thrombosis within the aneurysmal sac. They also noted that treatment and management, including anticoagulant therapy, for asymptomatic PVAs remained unclear. Previous literature reported that our indications for treating PVAs are as follows: 1) all symptomatic VAs, 2) asymptomatic saccular shaped and large (>20 mm) fusiform-type VAs, and 3) VAs with thrombosis. According to these strategies, we have already treated some PVA cases with no severe complications. Even though there may be some differences between PVAs and SVAs, we can consider SVA a deep venous aneurysm like PVA; therefore, we define the surgical indication for SVA, such as symptomatic VAs, saccular typed, and large-sized (more than 20 mm) fusiform-shaped VAs, and VAs containing thrombi, the same as the indication for PVA. The SVA, in this case, showed asymptomatic but saccular-shaped and thrombi-containing VA; thus, we treated this SVA with surgical excision. Even though there may be some differences between PVAs and SVAs, we could treat this case with SVA successfully, with no complications, using the same treatment strategies as for PVAs. We should note that the SV is also part of the deep venous system, and that it can be a cause of VTE, especially PE. Thus, our application of surgical treatment, in this case, was acceptable.

There is no doubt that surgical management is the gold standard for treating lower extremity VAs.^2)^ Even though surgical treatment is indicated for lower extremity VAs, anticoagulants alone are inadequate for patients with VAs because of the 80% risk of PE occurrence.^7)^ Calligaro et al.^4)^ also reported that most VAs in the lower extremities showed recurrent PE, despite non-operative medication with anticoagulant therapy. The VAs in the lower extremities, especially in the PV, can carry a high risk of PE; thus, surgical management is warranted. In this case, we treated VA in the SV by surgical excision, due to the risk of PE. In comparison to the rate of DVT between the PV and SV, the popliteal vein is more prone to DVT than the SV.^8)^ However, the SV is connected directly to the PV; thus, DVT in the SV can extend to the PV. Therefore, we excised this SVA. The surgical procedures include ligation, tangential excision with lateral venorrhaphy, autogenous vein patchplasty, and total resection, with or without graft interposition.^9)^ For saccular-type VAs, we can easily conduct tangential aneurysmectomy with lateral venorrhaphy. However, for fusiform-type VAs, it is difficult to perform partial aneurysmectomy and vein plasty; thus, total resection, with end-to-end anastomosis or graft interposition, are recommended to preserve venous continuity. In our case, the VA was located in the SV; thus, we did not need to maintain venous continuity, and we only performed total excision for the VA. After the operation, this patient showed no symptoms of venous insufficiency, including lower limb edema.

## Conclusion

In conclusion, VA is an unusual vascular lesion that can lead to significant complications, especially PE. VA might be misdiagnosed; thus, we should always keep the possibility of VAs in mind when we find VTE. With regard to VAs in the lower extremities, in addition to PVAs, SVAs should be properly treated according to the size and form of the aneurysm and the presence of thrombus. Surgical repair is the best type of treatment to prevent PE in patients with VAs.

## Notes

We obtained the consent form from this patient to report this paper.
